# Supplementary material for: A New, Validated GC-PICI-MS Method for the Quantification of 32 Lipid Fatty Acids via Base-Catalyzed Transmethylation and the Isotope-Coded Derivatization of Internal Standards
Source: Metabolites. 2025 Feb 7;15(2):104. doi: 10.3390/metabo15020104 (PMC11857457; doi:10.3390/metabo15020104)
Supplement: Supplementary file 1 [file metabolites-15-00104-s001.zip › metabolites-3445239-supplementary.pdf]

Table S1. List of lipids used in the study of the transmethylation reaction yields.

Lipid	Abbreviation	Molar Mass [g/mol]	Concentration [nmol/mL]
1-hexadecanoyl-rac-glycerol	MG 16:0	330.5	10.0
1,2-dihexadecanoyl-rac-glycerol	DG 16:0/16:0	568.9	5.0
1,2,3-trihexadecanoyl-sn-glycerol	TG 3x16:0	807.3	3.3
1,2-diheptadecanoyl-sn-glycero-3-phosphate	PA 17:0/17:0	698.9	5.0
1,2-ditetradecanoyl-sn-glycero-3-phosphocholine	PC 14:0/14:0	677.9	5.0
1,2-dihexadecanoyl-sn-glycero-3-phosphoethanolamine	PE 16:0/16:0	692.0	5.0
1,2-di-(9Z-octadecenoyl)-sn-glycero-3-phosphoethanolamine	PE 18:1/18:1	744.0	5.0
1,2-di-(9Z-octadecenoyl)-sn-glycero-3-phospho-(1'-sn-glycerol)	PG 18:1/18:1	796.5	5.0
1,2-ditetradecanoyl-sn-glycero-3-phosphoserine	PSer 14:0/14:0	701.8	5.0
1-(9Z-octadecenoyl)-sn-glycero-3-phosphate	LysoPA 18:1	458.2	10.0
2-tetradecanoyl-sn-glycero-3-phosphoethanolamine	LysoPE 14:0	425.5	10.0
1-octadecanoyl-sn-glycero-3-phospho-(1'-sn-glycerol)	LysoPG 18:0	534.6	10.0
1-(9Z-heptadecenoyl)-glycero-3-phosphoserine	LysoPSer 17:1	531.6	10.0
Cholest-5-en-3β-yl octadecanoate	CHE 18:0	653.1	10.0
O-hexadecanoyl-R-carnitine	Carnitine 16:0	323.9	10.0
N-(heptadecanoyl)-sphing-4-enine-1-phosphocholine	SM 17:0	717.1	10.0
N-(dodecanoyl)-sphing-4-enine	Ceramide 12:0	481.8	10.0
N-(dodecanoyl)-1-β-glucosyl-sphing-4-enine	GluCer 12:0	436.1	10.0

Table S2. Concentration of the investigated 32 FAME standards analyzed in calibration levels.

FAME	Concentration [μg/mL]												
	Original	Level 1	Level 2	Level 3	Level 4	Level 5	Level 6	Level 7	Level 8	Level 9	Level 10	Level 11	Level 12
C8:0	416.4	93.7	46.8	23.4	11.71	5.86	2.928	1.464	0.732	0.366	0.1830	0.0915	0.0457
C10:0	410.6	92.4	46.2	23.1	11.55	5.77	2.887	1.444	0.722	0.361	0.1804	0.0902	0.0451
C12:0	416.1	93.6	46.8	23.4	11.70	5.85	2.926	1.463	0.731	0.366	0.1829	0.0914	0.0457
C13:0	207.6	46.7	23.4	11.7	5.84	2.92	1.460	0.730	0.365	0.182	0.0912	0.0456	0.0228
C14:0	415.7	93.5	46.8	23.4	11.69	5.85	2.923	1.461	0.731	0.365	0.1827	0.0913	0.0457
C14:1(n-5)	207.4	46.7	23.3	11.7	5.83	2.92	1.458	0.729	0.365	0.182	0.0911	0.0456	0.0228
C15:0	206.5	46.5	23.2	11.6	5.81	2.90	1.452	0.726	0.363	0.181	0.0907	0.0454	0.0227
C16:0	622.7	140.1	70.1	35.0	17.51	8.76	4.378	2.189	1.095	0.547	0.2736	0.1368	0.0684
C16:1(n-7)	211.8	47.7	23.8	11.9	5.96	2.98	1.489	0.745	0.372	0.186	0.0931	0.0465	0.0233
C17:0	198.9	44.8	22.4	11.2	5.59	2.80	1.399	0.699	0.350	0.175	0.0874	0.0437	0.0219
C17:1(n-7)	212.7	47.9	23.9	12.0	5.98	2.99	1.496	0.748	0.374	0.187	0.0935	0.0467	0.0234
C18:0	417.6	94.0	47.0	23.5	11.75	5.87	2.936	1.468	0.734	0.367	0.1835	0.0918	0.0459
C18:1(n-9)t	198.5	44.7	22.3	11.2	5.58	2.79	1.396	0.698	0.349	0.174	0.0872	0.0436	0.0218
C18:1(n-9)	417.7	94.0	47.0	23.5	11.75	5.87	2.937	1.468	0.734	0.367	0.1836	0.0918	0.0459
C18:2(n-6)t	202.8	45.6	22.8	11.4	5.70	2.85	1.426	0.713	0.356	0.178	0.0891	0.0446	0.0223
C18:2(n-6)	210.1	47.3	23.6	11.8	5.91	2.95	1.477	0.739	0.369	0.185	0.0923	0.0462	0.0231
C18:3(n-6)	208.5	46.9	23.5	11.7	5.86	2.93	1.466	0.733	0.367	0.183	0.0916	0.0458	0.0229
C18:3(n-3)	209.0	47.0	23.5	11.8	5.88	2.94	1.470	0.735	0.367	0.184	0.0918	0.0459	0.0230
C20:0	422.2	95.0	47.5	23.7	11.87	5.94	2.969	1.484	0.742	0.371	0.1855	0.0928	0.0464
C20:1(n-9)	208.1	46.8	23.4	11.7	5.85	2.93	1.463	0.732	0.366	0.183	0.0915	0.0457	0.0229
C20:2(n-6)	208.2	46.8	23.4	11.7	5.86	2.93	1.464	0.732	0.366	0.183	0.0915	0.0457	0.0229
C21:0	207.9	46.8	23.4	11.7	5.85	2.92	1.462	0.731	0.365	0.183	0.0914	0.0457	0.0228
C20:3(n-6)	214.3	48.2	24.1	12.1	6.03	3.01	1.507	0.753	0.377	0.188	0.0942	0.0471	0.0235
C20:4(n-6)	206.3	46.4	23.2	11.6	5.80	2.90	1.451	0.725	0.363	0.181	0.0907	0.0453	0.0227
C20:3(n-3)	198.9	44.8	22.4	11.2	5.59	2.80	1.399	0.699	0.350	0.175	0.0874	0.0437	0.0219
C22:0	414.7	93.3	46.7	23.3	11.66	5.83	2.916	1.458	0.729	0.364	0.1822	0.0911	0.0456
C22:1(n-9)	209.1	47.0	23.5	11.8	5.88	2.94	1.470	0.735	0.368	0.184	0.0919	0.0459	0.0230
C20:5(n-3)	190.7	42.9	21.5	10.7	5.36	2.68	1.341	0.670	0.335	0.168	0.0838	0.0419	0.0210
C23:0	197.2	44.4	22.2	11.1	5.55	2.77	1.387	0.693	0.347	0.173	0.0867	0.0433	0.0217
C24:0	418.3	94.1	47.1	23.5	11.76	5.88	2.941	1.471	0.735	0.368	0.1838	0.0919	0.0460
C24:1(n-9)	208.5	46.9	23.5	11.7	5.86	2.93	1.466	0.733	0.367	0.183	0.0916	0.0458	0.0229
C22:6(n-3)	209.1	47.0	23.5	11.8	5.88	2.94	1.470	0.735	0.368	0.184	0.0919	0.0459	0.0230

Table S3A. FAME calibration with the traditional single-IS GC-EI-SIM-MS method.

Single-IS	Concentration [µg/mL]				Precision (RSD [%])				Accuracy [%]				Ranges of quantification
EI-MS	LLOQ	low QC	medium QC	high QC	LLOQ	low QC	medium QC	high QC	LLOQ	low QC	medium QC	high QC	[µg/mL]
**C8:0**	0.183	0.370	11.7	23.5	3.37	4.22	2.13	0.99	103	127	106	88	0.183 - 23.5
**C10:0**	0.180	0.360	5.8	23.5	1.64	1.21	1.85	13.36	81	103	100	89	0.180 - 23.5
**C12:0**	0.180	0.370	11.7	23.5	3.03	0.19	0.10	0.03	92	118	122	85	0.180 - 23.5
**C13:0**	0.046	0.091	2.9	23.3	6.88	2.43	1.04	0.34	89	92	107	90	0.046 - 23.3
**C14:0**	0.183	0.370	5.8	23.2	1.99	1.34	0.65	0.43	121	106	101	87	0.183 - 23.2
**C14:1(n-5)**	0.182	0.360	5.8	23.3	0.53	1.33	0.35	0.40	91	97	103	93	0.182 - 23.3
**C15:0**	0.023	0.045	2.9	23.2	13.93	2.66	0.83	0.00	104	94	104	94	0.023 - 23.2
**C16:0**	0.273	0.550	17.5	35.5	4.23	2.03	0.79	0.32	88	109	117	88	0.273 - 35.5
**C16:1(n-7)**	0.047	0.093	3.0	47.7	7.57	2.37	0.95	0.21	86	98	102	94	0.047 - 47.7
**C17:0**	0.044	0.087	2.8	22.5	0.21	3.56	0.34	1.13	80	80	97	97	0.044 - 22.4
**C17:1(n-7)**	0.047	0.093	3.0	24.0	3.09	3.52	0.18	0.36	115	105	96	96	0.047 - 24.0
**C18:0**	0.092	0.184	5.9	93.9	2.76	3.39	1.11	1.13	101	85	99	97	0.092 - 93.9
**C18:1(n-9)t**	0.022	0.044	2.8	44.7	8.79	7.21	0.03	1.28	90	96	106	93	0.022 - 44.7
**C18:1(n-9)**	0.092	0.184	5.9	47.5	1.13	9.73	0.49	0.26	105	76	89	102	0.092 - 47.5
**C18:2(n-6)t**	0.180	0.360	5.7	45.6	6.68	0.81	2.54	0.47	103	85	94	99	0.180 - 45.6
**C18:2(n-6)**	0.180	0.370	5.9	47.3	6.00	6.84	0.74	0.84	106	80	89	100	0.180 - 47.3
**C18:3(n-6)**	0.180	0.370	5.9	23.5	1.74	2.39	0.19	0.94	101	92	100	96	0.180 - 23.5
**C18:3(n-3)**	0.092	0.180	2.9	47.0	4.40	2.02	5.13	2.52	116	210	76	102	0.092 - 47.0
**C20:0**	0.186	0.370	11.9	94.5	0.75	1.37	0.44	2.84	102	86	93	99	0.186 - 94.5
**C20:1(n-9)**	0.180	0.370	5.9	46.8	0.43	8.54	1.61	2.44	110	88	91	101	0.180 - 46.8
**C20:2(n-6)**	1.460	2.900	12.0	46.8	3.04	1.54	0.15	1.54	87	90	100	100	1.460 - 46.8
**C21:0**	0.180	0.370	5.8	46.8	0.78	3.02	0.71	2.90	116	83	87	101	0.180 - 46.8
**C20:3(n-6)**	0.047	0.094	3.0	24.1	6.74	1.94	0.35	1.87	111	81	91	95	0.047 - 24.1
**C20:4(n-6)**	0.045	0.091	2.9	23.2	1.51	9.17	14.44	13.50	82	73	115	83	0.045 - 23.2
**C20:3(n-3)**	0.087	0.170	5.6	45.0	5.25	3.93	1.22	6.29	85	70	89	99	0.087 - 45.0
**C22:0**	0.182	0.360	11.7	93.3	0.99	2.60	1.46	0.24	97	77	91	100	0.182 - 93.3
**C22:1(n-9)**	0.370	1.700	5.9	47.1	1.05	1.50	2.03	2.73	103	81	86	102	0.370 - 47.1
**C20:5(n-3**	0.084	0.170	5.4	21.5	10.41	4.77	1.09	1.88	102	74	102	90	0.084 - 21.5
**C23:0**	0.170	0.350	2.8	11.1	0.58	3.05	2.59	1.82	102	70	89	109	0.170 - 11.1
**C24:0**	0.370	0.740	11.8	94.1	1.26	2.30	0.65	0.21	122	75	84	99	0.370 - 94.1
**C24:1(n-9)**	1.470	2.900	12.0	46.9	5.66	3.81	2.80	4.49	77	78	93	103	1.470 - 46.9
**C22:6(n-3)**	0.092	0.180	5.9	47.4	0.64	4.02	1.29	1.99	86	56	77	101	0.092 - 47.4

The concentration calibration range for each FAME analyte was estimated using calibration points corresponding to an R2 ≥ 0.985.

Table S3B. FAME calibration with the new isotope-coded multi-IS GC-EI-SIM-MS method.

IC-Multi-IS	Concentration [µg/mL]				Precision (RSD [%])				Accuracy [%]				Ranges of quantification
EI-MS	LLOQ	low QC	medium QC	high QC	LLOQ	low QC	medium QC	high QC	LLOQ	low QC	medium QC	high QC	[µg/mL]
**C8:0**	0.183	0.731	11.7	93.7	1.91	3.03	5.39	2.78	96	120	115	100	0.183 - 93.7
**C10:0**	0.180	0.721	5.8	92.4	1.41	1.95	3.88	6.05	95	120	114	92	0.180 - 92.4
**C12:0**	0.182	0.731	2.9	2.3	1.81	1.98	4.19	2.16	100	118	121	90	0.180 - 2.3
**C13:0**	0.046	0.182	1.5	11.7	3.19	2.20	4.37	1.74	81	109	113	93	0.046 - 11.7
**C14:0**	0.365	0.183	2.9	12.2	1.84	2.30	4.13	1.77	100	123	131	86	0.365 - 12.2
**C14:1(n-5)**	0.046	0.182	0.7	5.8	3.94	3.37	2.48	4.94	85	109	118	90	0.046 - 5.8
**C15:0**	0.045	0.180	1.5	11.6	2.24	2.65	4.60	1.79	82	111	119	90	0.045 - 11.6
**C16:0**	0.273	1.090	4.4	35.0	1.21	2.01	2.36	1.48	103	145	120	87	0.273 - 35.0
**C16:1(n-7)**	0.046	0.186	1.5	11.9	2.83	4.31	3.71	1.53	83	110	116	91	0.047 - 11.9
**C17:0**	0.084	0.174	1.4	11.2	2.05	2.93	5.52	1.88	96	109	114	89	0.084 - 11.2
**C17:1(n-7)**	0.093	0.373	1.5	12.0	1.43	2.55	4.43	1.59	121	125	88	120	0.093 - 12.0
**C18:0**	0.183	0.367	2.9	93.9	0.78	2.53	2.91	3.32	96	116	138	96	0.183 - 93.9
**C18:1(n-9)t**	n.a.												
**C18:1(n-9)**	0.183	0.734	2.9	11.7	4.53	3.28	3.09	9.96	97	120	115	87	0.183 - 11.7
**C18:2(n-6)t**	0.022	0.089	1.4	11.4	7.26	9.02	4.67	2.89	92	99	106	87	0.022 - 11.4
**C18:2(n-6)**	0.023	0.092	1.5	11.8	3.97	9.77	4.11	4.59	84	94	110	93	0.023 - 11.8
**C18:3(n-6)**	0.183	0.733	1.5	11.7	5.45	3.99	4.45	1.26	125	126	123	86	0.183 - 11.7
**C18:3(n-3)**	0.023	0.091	1.5	11.8	7.16	6.10	4.16	1.98	87	92	101	97	0.023 - 11.8
**C20:0**	0.186	0.742	3.0	47.3	1.43	2.06	4.74	10.24	93	120	119	88	0.186 - 47.3
**C20:1(n-9)**	0.091	0.365	1.5	11.7	2.35	1.61	5.51	2.08	119	111	109	93	0.091 - 11.7
**C20:2(n-6)**	0.182	0.731	1.5	11.7	7.46	4.39	7.33	1.48	106	103	100	97	0.182 - 11.7
**C21:0**	0.040	0.182	1.5	11.7	8.84	2.89	5.00	1.92	92	91	99	98	0.040 - 11.7
**C20:3(n-6)**	n.a.												
**C20:4(n-6)**	n.a.												
**C20:3(n-3)**	0.174	0.699	1.4	22.4	12.33	7.41	7.41	11.86	106	90	90	86	0.174 - 22.4
**C22:0**	0.046	0.182	2.9	23.3	8.58	3.31	5.09	1.71	107	92	105	96	0.046 - 23.3
**C22:1(n-9)**	n.a.												
**C20:5(n-3**	n.a.												
**C23:0**	0.021	0.086	1.4	11.1	15.37	11.05	5.78	2.14	89	99	104	96	0.021 - 11.1
**C24:0**	n.a.												
**C24:1(n-9)**	0.046	0.183	1.5	11.7	5.16	7.99	14.24	20.07	100	92	110	94	0.046 - 11.7
**C22:6(n-3)**	n.a.												

n.a. = not available

The concentration calibration range for each FAME analyte was estimated using calibration points corresponding to an R2 ≥ 0.985.

Table S3C. FAME calibration with the traditional single-IS GC-PICI-SIM-MS method.

Single-IS	Concentration [µg/mL]				Precision (RSD [%])				Accuracy [%]				Ranges of quantification
PICI-MS	LLOQ	low QC	medium QC	high QC	LLOQ	low QC	medium QC	high QC	LLOQ	low QC	medium QC	high QC	[µg/mL]
**C8:0**	0.091	0.370	2.9	93.4	5.01	2.49	2.95	2.95	81	104	102	91	0.091 - 93.4
**C10:0**	0.090	0.360	2.9	92.4	4.35	2.21	6.28	3.71	81	103	100	89	0.090 - 92.4
**C12:0**	0.180	0.731	2.9	93.6	7.14	7.09	7.17	2.31	88	102	98	88	0.180 - 93.6
**C13:0**	0.023	0.091	1.5	46.7	1.97	4.61	8.46	1.44	88	106	95	89	0.023 - 46.7
**C14:0**	0.183	0.731	2.9	93.5	3.69	5.72	6.20	0.97	91	103	101	88	0.183 - 93.5
**C14:1(n-5)**	0.046	0.182	1.5	46.7	2.09	1.43	4.41	0.29	96	100	100	90	0.046 - 46.7
**C15:0**	0.023	0.091	1.5	23.2	2.24	4.92	4.13	2.45	83	101	101	92	0.023 - 23.2
**C16:0**	0.068	0.274	4.4	70.1	0.69	2.05	3.18	3.36	86	99	101	95	0.068 - 70.1
**C16:1(n-7)**	0.047	0.190	1.5	24.4	2.36	2.22	3.13	0.52	106	101	98	85	0.047 - 24.4
**C17:0**	0.022	0.087	1.4	22.4	6.41	4.01	3.14	0.79	96	99	98	85	0.022 - 22.4
**C17:1(n-7)**	0.093	0.373	1.5	47.9	3.26	4.91	2.90	0.71	119	111	106	93	0.093 - 47.9
**C18:0**	0.046	0.184	2.9	47.5	2.07	1.30	2.67	0.62	97	100	99	86	0.046 - 47.5
**C18:1(n-9)t**	0.022	0.087	1.4	22.3	2.02	1.93	2.71	3.17	96	96	99	96	0.022 - 22.3
**C18:1(n-9)**	0.092	5.870	23.5	93.9	0.91	0.98	3.57	3.65	84	91	94	110	0.092 - 93.9
**C18:2(n-6)t**	0.022	0.350	2.9	23.5	1.70	3.78	1.88	0.30	98	100	93	93	0.022 - 23.5
**C18:2(n-6)**	0.023	0.092	1.5	24.3	4.68	2.43	2.48	0.26	112	95	94	89	0.023 - 24.3
**C18:3(n-6)**	0.023	0.091	1.5	23.5	1.22	1.74	1.65	0.40	106	106	91	89	0.023 - 23.5
**C18:3(n-3)**	0.023	0.091	1.5	24.5	2.57	2.07	2.57	0.44	119	96	94	90	0.023 - 24.5
**C20:0**	0.046	0.186	3.0	47.3	3.77	1.01	2.55	0.26	113	92	97	88	0.046 - 47.3
**C20:1(n-9)**	0.091	0.365	1.5	23.4	2.19	4.78	2.53	0.36	102	101	100	95	0.091 - 23.4
**C20:2(n-6)**	0.046	0.180	1.5	46.8	5.83	3.25	2.69	0.50	104	93	92	93	0.046 - 46.8
**C21:0**	0.023	0.092	1.5	23.4	3.36	1.51	2.43	0.26	120	95	93	90	0.023 - 23.4
**C20:3(n-6)**	0.047	0.190	1.5	24.1	3.72	0.87	2.87	0.47	106	92	92	91	0.047 - 24.1
**C20:4(n-6)**	0.725	1.450	2.9	23.2	2.89	3.56	2.26	0.48	81	80	93	96	0.725 - 23.2
**C20:3(n-3)**	0.699	2.797	1.4	22.4	1.45	1.03	3.80	0.54	86	96	86	93	0.699 - 22.4
**C22:0**	0.090	0.360	2.9	47.1	2.32	3.43	2.82	0.39	105	86	91	92	0.090 - 47.1
**C22:1(n-9)**	0.046	0.180	1.5	23.7	2.87	1.48	3.10	0.47	119	91	95	93	0.046 - 23.7
**C20:5(n-3**	0.042	0.170	1.3	21.5	2.39	1.15	0.98	0.45	120	85	90	92	0.042 - 21.5
**C23:0**	0.087	0.350	1.4	44.4	9.01	4.68	2.68	0.70	92	90	89	98	0.087 - 44.4
**C24:0**	0.092	11.760	24.0	94.1	2.92	8.05	8.83	1.21	86	97	100	103	0.092 - 94.1
**C24:1(n-9)**	0.180	0.733	1.5	46.9	5.49	2.28	4.03	1.56	92	93	89	103	0.180 - 46.9
**C22:6(n-3)**	0.023	0.092	1.5	24.3	6.88	5.63	2.65	0.74	117	94	91	92	0.023 - 24.3

The concentration calibration range for each FAME analyte was estimated using calibration points corresponding to an R2 ≥ 0.985.

Table S3D. FAME calibration with the new isotope-coded multi-IS GC-PICI-SIM-MS method.

IC-Multi-IS	Concentration [µg/mL]				Precision (RSD [%])				Accuracy [%]				Ranges of quantification
PICI-MS	LLOQ	low QC	medium QC	high QC	LLOQ	low QC	medium QC	high QC	LLOQ	low QC	medium QC	high QC	[µg/mL]
**C8:0**	0.046	0.183	2.9	93.4	2.95	3.36	2.95	2.99	112	96	94	99	0.046 - 93.4
**C10:0**	0.045	0.180	2.9	92.4	1.52	3.36	2.87	2.25	105	99	98	98	0.045 - 92.4
**C12:0**	0.180	0.360	2.9	93.6	2.91	2.63	2.85	1.62	86	80	98	97	0.180 - 93.6
**C13:0**	0.023	0.091	1.5	46.7	0.90	1.20	2.85	1.78	106	99	97	98	0.023 - 46.7
**C14:0**	0.046	0.183	2.9	93.5	1.43	2.11	2.81	1.19	82	94	100	97	0.046 - 93.5
**C14:1(n-5)**	0.023	0.091	1.5	46.7	2.74	2.51	2.85	1.56	115	100	98	97	0.023 - 46.7
**C15:0**	0.045	0.180	1.5	46.5	2.31	1.81	2.95	1.11	89	99	94	95	0.045 - 46.5
**C16:0**	0.137	0.550	4.4	140.1	0.79	1.57	2.97	1.27	82	98	105	94	0.137 - 140.1
**C16:1(n-7)**	0.023	0.093	1.5	47.7	4.97	3.37	2.94	1.20	105	97	97	98	0.023 - 47.7
**C17:0**	0.044	0.170	1.4	44.8	1.10	1.79	3.14	0.87	85	99	106	92	0.044 - 44.8
**C17:1(n-7)**	0.047	0.190	1.5	47.9	3.94	4.69	3.04	1.16	120	104	99	96	0.047 - 47.9
**C18:0**	0.046	0.184	2.9	93.9	1.73	2.63	2.91	0.80	91	97	97	96	0.046 - 93.9
**C18:1(n-9)t**	0.022	0.087	1.4	44.7	1.78	3.14	3.01	10.15	87	98	101	98	0.022 - 44.7
**C18:1(n-9)**	0.046	0.183	2.9	93.9	5.43	3.38	2.99	6.68	84	102	99	96	0.046 - 93.9
**C18:2(n-6)t**	0.022	0.089	1.4	45.6	4.74	2.63	2.90	1.38	108	97	94	98	0.022 - 45.6
**C18:2(n-6)**	0.023	0.092	1.5	47.3	3.69	1.93	2.90	1.38	106	98	94	98	0.023 - 47.3
**C18:3(n-6)**	0.023	0.091	1.5	46.9	0.60	1.23	1.95	0.94	100	108	100	94	0.023 - 46.9
**C18:3(n-3)**	0.023	0.091	1.5	47.0	2.08	2.80	2.92	1.93	120	97	93	99	0.023 - 47.0
**C20:0**	0.046	0.186	3.0	94.5	3.20	2.50	3.07	0.21	90	95	99	94	0.046 - 94.5
**C20:1(n-9)**	0.047	0.180	1.5	46.8	0.48	1.96	3.06	0.79	122	98	98	95	0.047 - 46.8
**C20:2(n-6)**	0.023	0.091	1.5	46.8	6.71	5.64	3.25	0.80	113	98	93	98	0.023 - 46.8
**C21:0**	0.023	0.092	1.5	46.8	2.93	3.46	2.95	0.31	105	102	96	94	0.023 - 46.8
**C20:3(n-6)**	0.023	0.094	1.5	48.2	7.87	4.27	3.24	1.39	110	98	94	98	0.023 - 48.2
**C20:4(n-6)**	0.023	0.091	1.5	46.4	7.76	2.49	1.50	0.07	115	115	87	98	0.023 - 46.4
**C20:3(n-3)**	0.044	0.170	1.4	22.4	7.90	3.93	3.96	0.65	86	100	108	103	0.044 - 22.4
**C22:0**	0.046	0.182	2.9	93.3	8.32	4.76	3.33	1.14	87	89	102	90	0.046 - 93.3
**C22:1(n-9)**	0.046	0.180	1.5	47.1	15.00	3.80	1.81	0.88	104	85	85	101	0.046 - 47.1
**C20:5(n-3**	0.042	0.170	1.3	21.5	9.42	6.15	2.68	0.14	109	99	94	94	0.042 - 21.5
**C23:0**	0.087	0.350	1.4	11.1	10.00	8.00	3.49	1.98	85	113	114	85	0.087 - 11.1
**C24:0**	0.046	7.400	24.0	94.1	5.54	12.80	0.54	0.26	108	85	95	108	0.046 - 94.1
**C24:1(n-9)**	0.023	0.092	1.5	46.9	5.69	8.21	4.35	15.00	104	87	99	98	0.023 - 46.9
**C22:6(n-3)**	0.046	0.180	1.5	47.4	12.65	4.37	2.82	2.32	95	88	90	100	0.046 - 47.4

The concentration calibration range for each FAME analyte was estimated using calibration points corresponding to an R2 ≥ 0.985.
